# Multi-faceted potential of sophoridine compound's anti-arrhythmic and antioxidant effects through ROS/CaMKII pathway

**DOI:** 10.1016/j.heliyon.2024.e37542

**Published:** 2024-09-06

**Authors:** Shuai Sun, Fangdi Shi, Gang Zhao, Hong Zhang

**Affiliations:** Department of Cardiology, Shanxi Provincial People's Hospital, Taiyuan, 030001, China

**Keywords:** Sophoridine, Anti-arrhythmia, Anti-oxidant, Zebrafish, ROS

## Abstract

Cardiac arrhythmias remain a significant cause of mortality and morbidity, for novel antiarrhythmic therapies. This study states that the first report of sophoridine (SPN), a quinolizidine alkaloid derived from traditional Chinese herbs, shows promise as a potential candidate due to its anti-arrhythmic and antioxidant properties. The study found that cell viability in H9C2 rat cardiomyocytes remained stable even when treated with SPN at a higher dosage of 100 μg/ml. This phenomenon was accompanied by increases in mitochondria-derived reactive oxygen species (ROS) and calcium/calmodulin-dependent protein kinase II (CaMKII) signaling, at 50 and 100 μg/ml. Glucose fluctuations regulate ventricular arrhythmias caused by SPN by activating the ROS/CaMKII pathway. Experimental models using zebrafish provided additional evidence supporting the regulatory effects of SPN on heart rate. In addition, the administration of SPN resulted in substantial deregulation of crucial genes involved in heart development (nppa, nppb, tnnt2a) at the transcriptional level in zebrafish. These findings provide insight into the various pharmacological properties of SPN and this opens up new possibilities for anti-arrhythmic treatment strategies.

## Highlights

• Sophoridine (SPN) is a promising antiarrhythmic derived from traditional Chinese herbs due to its multifaceted pharmacological properties.
• SPN has anti-arrhythmic and antioxidant properties that may help treat cardiac arrhythmias and oxidative stress.
• SPN treatment increases ROS and CaMKII signaling, suggesting that the ROS/CaMKII pathway is involved in SPN's action.
• In zebrafish models, SPN regulates heart rate, demonstrating its potential as a cardiac modulator across species.

## Graphical abstract

**Figure:**
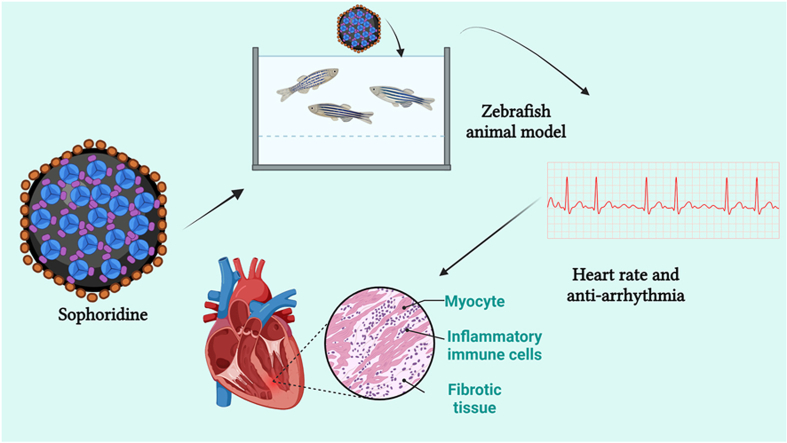

## 1 Introduction

Arrhythmia is a prevalent and hazardous cardiovascular condition. Research and development of anti-arrhythmic drugs have been continuous, although there has been no significant advancement in the last two decades. Malignant arrhythmias are responsible for 88 % of sudden cardiac fatalities in China [1,2]. Antiarrhythmic Drugs (AADs) are administered to stop atrial and ventricular arrhythmias and prevent their recurrence [3]. Arrhythmias can be categorized based on the specific point in the conduction channel where they occur [4]. Anti-arrhythmic medicines are membrane-active medications that regulate ion channel activity, alter membrane pump function, and activate or inhibit membrane receptors [5]. Various techniques can be used to evaluate heart functioning in zebrafish embryos, such as stopwatch counting, micro pressure system, Laser Doppler microscope technique, and electrocardiogram [6]. Many antiarrhythmic treatments include calming Chinese herbal ingredients including amber in Wenxin Keli, magnolia berry (*Schisandra chinensis*), jujube (*Ziziphus jujube*), and oyster in the *Yangxin pingmai* decoction [7]. Sophoridine is a bioactive quinolizidine alkaloid extracted from the leaves of the Leguminous plant *Sophora alopecuroides*. Accumulating evidence shows that Sophoridine has significant pharmacological effects on inflammatory illnesses, infectious diseases, and malignancies [8]. Ischemia and reperfusion to the heart result in energetic stress and oxidative stress, which can cause apoptotic and necrotic cell death [9]. Sophoridine is a quinolizidine alkaloid obtained from *Sophora alopecuroides* L., commonly utilized in Chinese traditional medicine. It demonstrates anticancer and anti-inflammatory properties as well as strong antiviral effects [10]. Sophoridine is said to possess anti-tumor and anti-viral properties. A study proposed that sophoridine improves LPS-induced inflammation by decreasing IL levels [11]. Sophoridine has hepatoprotective properties and modulates PI3K pathway activity to decrease the levels of inflammatory mediators and oxidative stress [10]. High-speed video imaging is utilized to analyze heart rate variability and rhythm by examining blood cell velocity using digital motion analysis. It is also used to assess heartbeat regularity by capturing flowing blood images in the caudal vasculature of zebrafish [12]. Cardiac arrhythmia, which is an irregularity in the heartbeat, is linked to the cardiotoxic effects of medications and sudden cardiac death [6]. Investigators explored the effects of various concentrations of sophoridine (10, 20, and 40 mM) on nerves within a permanent middle cerebral artery occlusion (PMCO) model. They found that sophoridine demonstrated promising neuroprotective properties, evidenced by diminished cerebral edema and infarct volume. These effects may be attributed to the downregulation of TRAF6 expression and the enhancement of erk 1/2 phosphorylation [13]. Calcium/calmodulin-dependent protein kinase II (CaMKII), a crucial serine/threonine protein kinase, is known to have a significant impact on cardiac electrophysiology, cardiac contraction, and calcium management. Research findings indicate that oxidized CaMKII (ox-CaMKII) is primarily activated through alteration of the regulatory domain methionine as a result of ROS generation [14]. From a scientific perspective, SPN has demonstrated numerous therapeutic benefits, including cytotoxic, anti-inflammatory, anti-asthma, anti-anaphylaxis, antimicrobial, antiviral, antiarrhythmic, and anti-fibrotic properties [15]. Cardiac arrhythmias impact approximately 2 % of persons living in the community, with an annual incidence of around 0.5 %. Arrhythmias can appear as harmless conditions like atrial and ventricular premature beats, or as serious arrhythmias as ventricular tachycardia (VT) and ventricular fibrillation (VF), which has effect in sudden cardiac death (SCD), responsible for 10–15 % of all fatalities [16]. There have been recent concerns raised about the safety of antiarrhythmic drugs when used in clinical settings. Limitations, such as adverse reactions and arrhythmogenic effects, are present in many drugs. New and improved antiarrhythmic medication development and marketing are currently moving at a pace of snail. Research into the development of effective and safe drugs or compounds to treat arrhythmia [17]. The study showed that our technique can evaluate cardiac physiology in zebrafish by measuring heart rate and rhythmicity through ROS/CaMKII pathway. Integrating emerging antiarrhythmic drugs and therapeutic strategies with advancements in novel antithrombotic agents, supported by computational modeling approaches, holds significant promise for enhancing outcomes in patients with atrial fibrillation and this will serve as a framework for future research.

## 2 Materials and methods

### 2.1 Characterization of SPN

The crystallinity of sophoridine residues was analyzed using X-ray diffractometry (Ultima IV, Japan) with copper Kα radiation, a voltage of 40 kV, an electrical current of 30 mA, and continuous scanning Angle from 10° to 80°. The crystallinity index (CI) is calculated using the formula: CI% = [(I002 − Iam)/I002] × 100.

I002 represents the greatest intensity at 2θ, 23° for the (002) lattice diffraction, while Iam represents the intensity at 2θ, 19° for the amorphous diffraction. The functional groups of SPN residues were identified using FTIR analysis conducted with a Thermo Scientific Nicolet IS5 instrument in the USA. The SPN residues were dried in an oven at 60 °C for 24 h and then combined with dry KBr. FTIR experiments were conducted within a spectrum range of 4000–400 cm^−1^.

### 2.2 Anti-oxidant activity on SPN

#### 2.2.1 ABTS scavenging activity

A mixture containing 10 ml of DPPH (98 %, Chem src, China) (7 mM ABTS, 2.45 mM K2S2O8) and 20 ml of methanol with a higher concentration of 75 μg/ml (SPN was utilized as a tested sample. The reactivity of different amounts of each solvent extract was compared to that of ascorbic acid. Each measurement was conducted a minimum of three times. ABTS scavenging percentage was determined for various concentrations (25, 50, and 75 μg/ml) for 45 min of SPN and a standard compound using the provided formula.

$$\text{ABTS}\;\%\;\text{Scavenging}=\frac{\text{Acontrol}\;-\;\text{Asample}}{\text{Acontrol}}\times 100$$

#### 2.2.2 DPPH activity

DPPH radical scavenging activity was assessed with minor adjustments. The reaction mixture contained 0.5 ml of SPN, 3 ml of methanol, and 0.3 ml of 0.5 mM DPPH radical solution in methanol. Ascorbic acid was used as a positive control. The absorbance was measured at 517 nm after incubating for 45 min using a spectrophotometer. We used the following equation to find the antioxidant activity.

$$\%\;\text{inhibition}=\frac{\left(A\;\text{control}\;-\;A\;\text{sample}\right)}{A\;\text{control}}\times 100$$

At the time of solution production, the absorbance of the control was measured (A_control_). The sample's absorbance as measured 45 min later [18].

### 2.3 Cell culture

Sourced from the cell bank of the Chinese academy of sciences, Shanghai, China. H9c2 cells were adopted. The cells were grown in Dulbecco's Modified Eagle Medium (DMEM), a high-glucose medium that contains 10 % fetal bovine serum (FBS), 4.5 g/L glucose, and 100 units/ml penicillin/streptomycin. The cultures were kept at 37 °C in a humidified environment with 5 % CO_2_.

### 2.4 Cell viability assay (MTT)

The MTT test was used to find out the viable cells. H9c2 cells were seeded into 96-well plates at a density of 7000 cells/well for the first 24 h of treatment and 5000 cells/well for 48 h. At 24 and 48 h, cells were subjected to varying concentrations of SPN compounds, ranging from 25, 50, 75, and 100 μg/ml. After that, for an extra 4 h, Then, 20 μl of MTT (3-(4,5-dimethylthiazol-2-yl)-2,5-diphenyl tetrazolium bromide, 5 mg/ml) from Sigma-Aldrich in Saint Louis, Missouri, USA was added. Following the removal of the medium, 200 μl of DMSO was added to every well to help dissolve the formazan crystals. Consumption measurements were taken using a micro plate reader (Epoch 2, Biotek, USA) calibrated to 545 nm as the final step.

### 2.5 Assessment of nuclei using DAPI analysis

DAPI and nucleic acid stains that can penetrate cells are suitable for examining nuclear structure and apoptotic fragments using fluorescence microscopy. To stain the nuclei, H9c2 cells were cultured with the specified medication dosage while maintaining a constant cell density of 2 × 10^4^ cells/well in 6-well culture plates. Following a 10-mins incubation in room temperature dark light and a rinse with PBS, the cell nuclei were seen under a 40x magnification lens by fluorescent microscopy (Olympus Optical, Tokyo, Japan).

### 2.6 Evaluation of ROS

The ROS levels were measured using a ROS assay kit (50101ES01, YEASEN, China). After resuspending 100,000 to 600,000 cells, which had been previously treated with drugs and culture media, a 10 μl of DCFH-DA solution was added. The cells were incubated at 37 °C for 0.5–4 h subsequently centrifugation at 600×*g* for 3–4 min at 4 °C. Bring the cell concentration down to 1 × 10^6^ cells/mL in 1× buffer or complete media with 10 % FBS omitting phenol red. Put 100,000 stained cells/well into a 96-well microplate with a dark, transparent bottom. Use a fluorescence plate reader in endpoint mode at 485/535 nm to measure the plate right away, regardless of whether there are compounds, media, or buffer present.

### 2.7 Cell apoptosis through ROS/CaMKII pathway

Flow cytometry was used to identify apoptotic cells after propidium iodide labeling of DNA fragments. Cells incubated in a hypotonic phosphate-citrate buffer with a quantitative DNA-binding dye, such PI, can reportedly cause a sub-G1 peak to be observed, which indicates DNA fragmentation. Apoptotic cells that have lost their DNA will show less staining and can be seen on the histogram to the left of the G1 peak. A 24-well culture plate was used to first seed H9c2 cells. To cause cell damage, the cells were incubated with SPN for 24 h after being treated with 7.81–500 μg/ml for 2 h. The cells that were floating and adhering were mixed with 750 μL of a hypotonic buffer that contained 50 μg/ml PI in a solution of 0.1 % sodium citrate and 0.1 % Triton X-100, which were then treated at 4 °C in the dark overnight. The next step was to run the samples via a Becton Dickinson FACS flow cytometer. Using FACS, 104 events were obtained [19].

### 2.8 Animals maintenance

The zebrafish used in our investigation were of the wild type. Proper care and maintenance practices were guaranteed by following to the NIH guidelines for laboratory animal care and usage. The zebrafish were incubated in 10-Liter tanks with recirculating systems and exposed to a light-dark cycle of 14 h. You should expect to be fed twice a day during the week and once a day on the weekends. The water was carefully regulated to keep it on a steady state of temperature 28.5 °C and a pH level of 7.5. During the night, a breeding trap was used to hold two adult males and one adult female to facilitate breeding followed by retrieval and placement in an incubator set at 28.5 °C the subsequent morning. Upon reaching the age range of 12–15 weeks, the zebrafish were transferred to tanks and sustained on a diet of paramecium until the initiation of testing.

### 2.9 Drug treatment

Per OECD guideline No. 12 (OECD, 2023), zebrafish are to be handled. We looked at the effects of SPN on development at doses between 20 and 100 μM. The SPN solutions were mixed with water that contained 0.05 % DMSO (v/v) after being dosed individually. For each treatment group, 12 week-old animals were picked at random and put into one well of 24-well plates with SPN solution. A supplemental food pellet was formed by combining the medicine formulations. Multiple time intervals for doing the designated experiments. Osypka Medical GmbH's PACE100H electronic pacemaker was used to deliver the electrical stimulation. As the cathode, borosilicate glass micropipettes that were already obtained from World Precision Instruments were filled with 3 M potassium chloride and had a resistance that varied between 2 and 10 MΩ. As mentioned before, a micromanipulator was used under microscopic guidance to place the electrode close to the atrium or ventricle. Numerous frequencies, spanning 80 to 180 bpm, were applied to the heart chambers using voltages ranging from 1 to 12 V [20]. Electrical stimulation has caused the stress to generate arrhythmia. The following groups were segregated for the experiments as indicated Control group consisted of normal zebrafish, while the experimental groups were ES (Electrical Stimulation), ES + SPN at concentrations of 25 μg/ml, 50 μg/ml, and 100 μg/ml [21].

### 2.10 Measurement of MDA, ROS, and T-SOD measurement

We mixed 50 zebrafish (12 weeks) and rinsed them with water. After that, the animals were placed in a centrifuge tube and mixed with 500 μl of saline in an ice bath to make them homogeneous. Centrifuged at 2500 rpm for 10 min at 4 °C, the animal homogenate was spun. Upon collection of the supernatants, the levels of malondialdehyde (MDA), reactive oxygen species (ROS), and superoxide dismutase (SOD) enzyme activity were measured using commercial kits from Biotechnology in accordance with the instructions provided by the manufacturer. We used WST-8 from the T-SOD kit to conduct a color reaction, and we measured the absorbance at 450 nm. The thiobarbituric acid reaction, which produces red compounds, is the basis of the MDA kit. At a wavelength of 535 nm, the absorbance was measured [22], [23].

### 2.11 Studies assessing the impact on the cardiovascular structure

There was an equal amount of male mature wild-type zebrafish and female zebrafish 13–15 weeks old. 30 zebrafish were randomly chosen for anti-arrhythmia tests and administered 6 ml of SPN solutions at five doses. Green fluorescence was observed in zebrafish at 12 weeks of age when they were moved to individual 24-well plates to monitor heart structure every 24 h for a total of 96 h. The animal was subjected to medications to evaluate stress and anxiety, which caused an increase in heart rate. Heart rates (beats per minute, bpm) were measured by counting the beats over a 20-s interval and then converting this count to beats per minute, either from a video recording or in real-time.

### 2.12 Behavioral tests

This study examined two behavioral endpoints in zebrafish: thigmotaxis and swimming activity during 1 h of free swimming. Initially, SPN was administered at doses of 0.25, 0.75, 1.5, 5, 10, 15, and 45 μM in the initial investigations. The zebrafish were individually taken out of their habitat the night before behavioral assessments and placed into 500 μL of water is added to each well of a 48-well plate. The fish were first exposed to light for 20 min to acclimate, and then they were moved to different solutions and kept in the light for an hour. The researchers used Zebrabox, made by ViewPoint Life Sciences in France, to record the fish's swimming activity. Dopaminergic antagonists were given to zebrafish after a 20-min acclimatization period in order to reduce malformations. Each fish's swimming distance was measured. To evaluate thigmotaxis, we measured the swim distances in the total and inner areas, and we set the thigmotaxis edge at 3 mm from the well wall. The distance ratio was calculated by dividing the total floating distance in the well area by the amount of distance floated in the inner zone. A lower ratio indicating increased thigmotaxis as indicative of elevated level.

### 2.13 RT-qPCR

A small number of zebrafish were present in each well of the twenty-four-well plates. Twelve zebrafish were chosen at random from the control and SPN-treated groups. Trizol (Accurate Biology, Wuhan, China) was used for mRNA extraction, and a NanoDrop 3300 Fluorospectrometer (Thermo Fisher Scientific, USA) was used for quantification. A constant range of 1.8–2.0 was maintained for the absorbance ratios at 260/280. Using the QuantiNova Reverse Transcription Kit from Qiagen, Germany, 1 μg of RNA was then used for complementary DNA synthesis. The standard methods for quantitative RT-qPCR were used. In the thermal cycling program, there was a denaturation phase lasting 2 min at 95 °C, and 15 s at 60 °C. To determine the levels of gene expression, relative quantification was used. The 2-△△Ct technique was used to compute the fold change after normalizing the raw Ct values to rpl13a, a housekeeping gene that was used to generate baseline conditions. Medication exposure verified the stability of the chosen housekeeping gene [24].

### 2.14 Histology and hematoxylin-eosin staining

Each batch of zebrafish (ten in total) was chosen at random from the control and drug-treated groups. There was an overnight period of immobilization at 4 °C for the animals after selection in a 4 % paraformaldehyde solution and subsequently rinsed thrice with phosphate-buffered saline for 5 min per rinse. Brain slices, 7 μm in thickness, were prepared by dehydration in an ethanol gradient, embedding in paraffin, and drying overnight at 37 °C. Subsequently, the sections underwent staining with hematoxylin-eosin, were then sectioned, and images were captured at a magnification of 200×.

#### 2.14.1 Statistical analysis

We used SPSS 26 (IBM SPSS, Chicago, IL, USA) to do all of our statistical analysis. We presented the data as the mean ± standard deviation. To compare the experimental groups, analysis of variance (ANOVA) was used, which was followed by post hoc LSD testing. We used Image J, version 1.4, from the National Institutes of Health (USA), to compare and analyze band intensities. At a significance level of p < 0.05, statistical significance was determined for the variances between the groups.

## 3 Results and discussion

### 3.1 Characterization of SPN

**Fig. 1:**
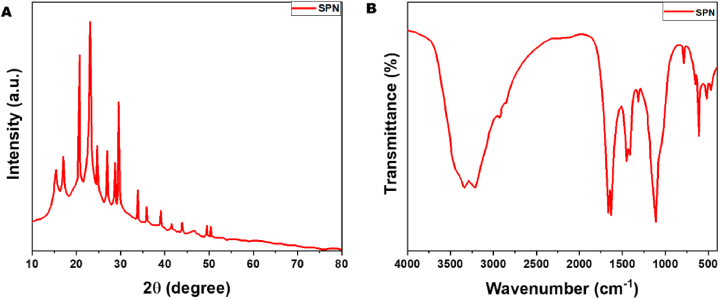
XRD pattern analysis of SPN (A) and FTIR spectrum of SPN (B).

The current work conducted XRD analysis on SPN, revealing a crystalline structure at 25.87⁰. The FTIR peaks displayed values of 1675 and 986.457, as depicted in Fig. 1(A and B). Also the three quinolizidine alkaloids have a common structural scaffold. Sophoridine is the C-5 epimer of matrine, and oxymatrine is its N-oxide derivative. The use of co-amorphous systems in the pharmaceutical industry is an exciting new direction. Diffraction peaks at 15.26⁰ and 20.93⁰ are observed in the crystalline form of sophoridine. Endothermic melting maxima are seen at 85.59 °C, 108.89 °C, and 265.05 °C for matrine, sophoridine, and resveratrol, respectively. At temperatures of 57.57 °C, 161.21 °C, and 205.12 °C, crystalline oxymatrine exhibits three endothermic peaks that have been reported [25]. The data reveal two distinct phases of sophoridine release from M-MSN–PNIPAAm. The initial release phase exhibited rapid kinetics, likely attributed to the swift diffusion of sophoridine across the surface of M-MSN–PNIPAAm [26].

### 3.2 Anti-oxidant activity on SPN

**Fig. 2:**
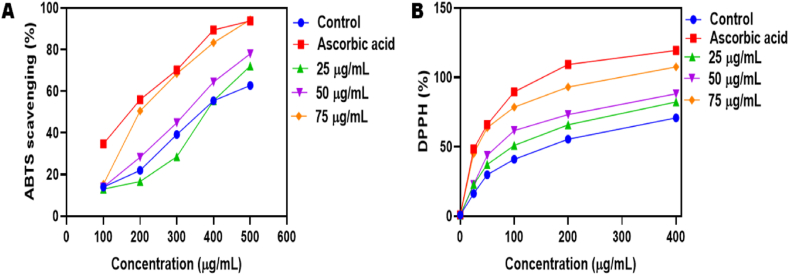
Antioxidant activity of SPN at 25, 50 and 75 μg/mL concentrations measured using ABTS (A) and DPPH scavenging assays for 45 min of incubation (B).

One way to measure a sample's antioxidant capacity is with the DPPH assay to decrease the stable free radical DPPH. Antioxidant activity of SPN was assessed at different doses of 25, 50 and 75 μg/mL at 45 min by ABTS and DPPH scavenging assays. Ascorbic acid is used as standard. The activity level of control, ascorbic acid, 25, 50 and 75 concentrations of SPN shows 62.79, 93.83, 72.09, 78.07 and 94.35 respectively for ABTS assay. Likewise, for DPPH assay the level is observed as 70.75, 119.47, 82.34, 88.13, 107.58 (Fig. 2). Similar quantified results observed in the root extract showed activity levels of 1.42 mg/ml in the CUPRAC reduction test, 2.45 mg/ml in the phosphomolybdenum test, 1.25 mg/ml in the ABTS test, and 8.41 mg/ml in the DPPH test [27]. Evaluating the antioxidant properties of methanolic extracts from different parts of *T. pallida* and comparing them to the standard BHT. TPL had the most significant activity among the extractives. The scavenging activities of TPL, TPRB, TPSB, and TPF were (91.05 % ± 1.10 %) at a concentration of 100 μg/ml. In comparison, the standard BHT exhibited a scavenging activity of 96.45 ± 0.41 % at the same concentration has been reported [28]. A dosage of 1000 μg/ml in the DPPH and MDA antioxidant assays showed less than 50 % efficacy in the in-vitro antioxidant activity for both the extracts and the four separate pure compounds (oxysophocarpine, oxymatrine, matrine, and sophoridine) The scavenging percentages of gallic acid, an established antioxidant, varied with concentration: 0.1 μg/ml - 15.48 %, and 0.5 μg/ml - 22.16 % [15].

### 3.3 Cell viability and DAPI staining

**Fig. 3:**
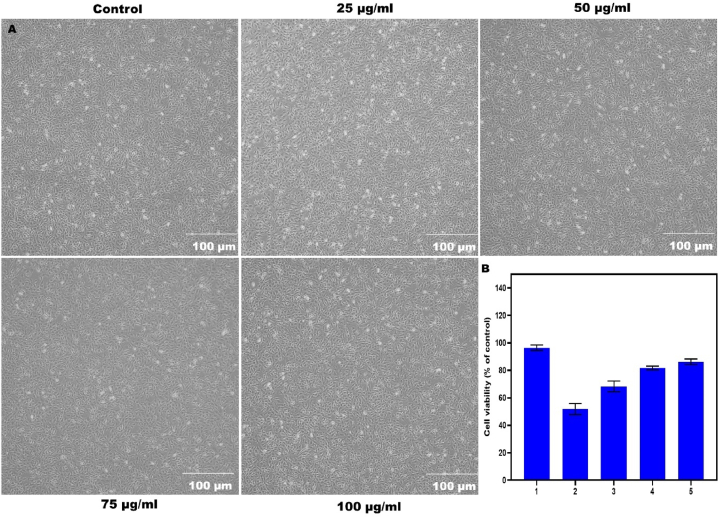
Phase contrast microscopic images on H9c2 cell viability of SPN concentrations at 25, 50, 75 and 100 μg/ml at 24 h of incubation (A); Cell viability % compared to control with statistical significance (B).

Phase contrast microscopy pictures were used to assess the viability of H9c2 cells treated with different concentrations of SPN, comparing cell viability percentages to the control group. No morphological alterations were seen after treatment with SPN at a dosage of 100 μg/ml at 10 min. Therefore, it demonstrates that the SPN does not have any detrimental impacts on cells (Fig. 3). Likewise, HBCD also causes cardiac arrhythmia in zebrafish. The exposure to HBCD led to SR Ca^2+^ excess in H9C2 cells. Deep sequencing was utilized to investigate the mechanisms of heart dysfunctions caused by HBCD has been reported [29]. Beyond 8 mM, no further cell death or growth was detected for either assay; a survival plateau was also noted. In the cell growth inhibition assay, around 30 % of H9c2 cells persisted even when administered DHA doses exceeding 10 mM. In the clonogenic test, tiny colonies that are viable are also revealed [30].

**Fig. 4:**
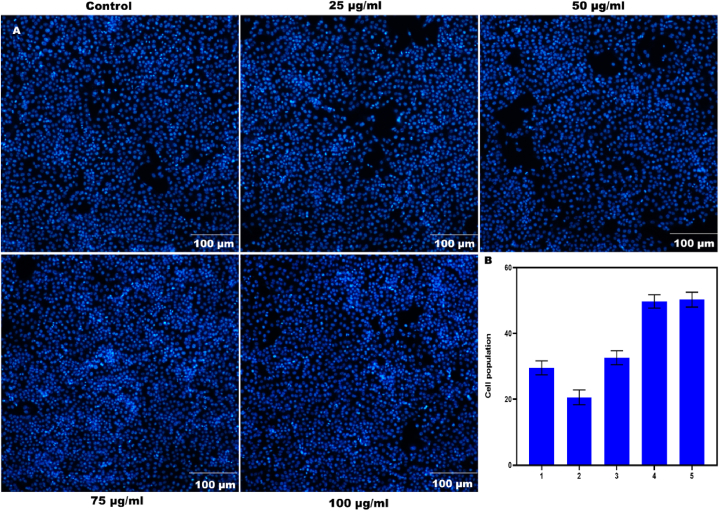
DAPI images demonstrate the nuclear structure of SPN-treated H9c2 cells at varying concentrations at 10 min. The trials were conducted three times (A). The cell population is displayed as a percentage, indicating its importance (B).

Apoptosis in cancer cells was induced and the cell development cycle's G1 and G2 stages were disrupted by sophoridine. Exposure to sophoridine led to inhibition of TOPO 1 enzyme activity in cancer cells. Findings indicated rapid absorption, distribution, and elimination of sophoridine without undergoing alterations via glomerular filtration. Morphological alterations included enlargement of mitochondria in S 180 sarcoma cells, cytoplasmic vacuolations in tumor cells, and a reduction in microvilli density on the cell membrane [31]. DAPI was used to visualize the nuclear organization of H9c2 cells treated with SPN at different doses. The cell population is represented as a percentage to show its significance (Fig. 4A and B). A similar outcome was reported in sophoridine affected BRL-3A cells in a concentration-dependent way. BRL3A cells exhibited nuclear alterations at 24 h, as indicated by DAPI staining [19]. Vericiguat (Veri) enhanced the survival of H9c2 cells subjected to hypoxia. Research has shown that after a myocardial infarction, the death of myocardial cells affects myocardial fibrosis, ventricular remodeling, and the control of cellular viability. Vericiguat improved left ventricular unfavourable remodeling and arrhythmias caused by myocardial infarction by affecting the CamkII signaling pathway [32].

### 3.4 Measurement of ROS and apoptosis ROS/CaMKII pathway

**Fig. 5:**
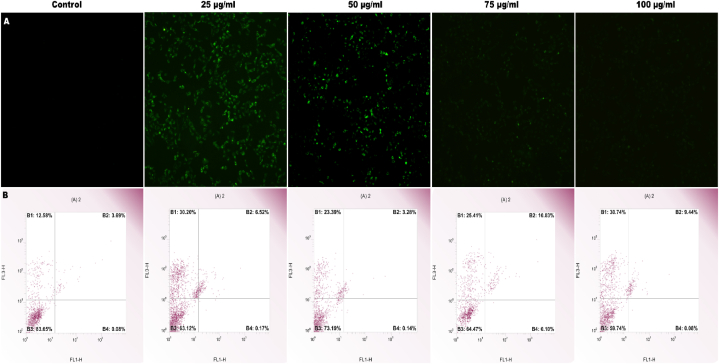
H9c2 cell lines were subjected to oxidative stress by exposure to various concentrations of SPN. Fluorescent microscope pictures were taken at 100 μm, 10× magnification (A). The percentage of apoptotic H9c2 cells was determined at different SPN doses by annexin V/PI staining (B).

The augmentation of SPN-mediated ROS and calcium/calmodulin-dependent protein kinase II (CaMKII) pathway activation facilitated the suppression of arrhythmias. Consequently, directing therapeutic interventions toward this signaling pathway could potentially serve as a novel treatment strategy in the future. Ventricular tachyarrhythmias may be caused by cardiac remodeling and mitochondrial oxidative stress, which lead to an abnormal coupling of Ca^2+^ and membrane potential (V m) via the NOX4/ROS/CaMKII pathway [33]. CaM-regulated protein kinases, including Ca^2^/CaM-dependent kinase II (CaMKII), have been identified as being dysregulated in many cardiac disorders. The multifaceted role of CaMKII, affecting both the function and expression of molecules involved in promoting and preventing irregular heart rhythms, complicates the assessment of the impact of the D129GCaM mutation on the entire organism [34]. Multiple lines of evidence point to ROS generation and the subsequent oxidative stress as causes of cell death, necrosis, and myocyte loss in the heart constituting a fundamental mechanism underlying doxorubicin-induced cardiotoxicity. H9c2 cell lines were exposed to different doses of SPN to induce oxidative stress, as shown in Fig. 5A. Equally, to detect reactive oxygen species (ROS), zebrafish larvae at 96 h post-fertilization were treated with 5 μM of 2,7-dichlorodihydrofluorescein diacetate (Cayman Chemical) for 40 min in the absence of light. We intended to confirm if the cardioprotective effects of medications used to treat heart disease in humans can be reproduced in zebrafish, in order to justify their suitability as a pharmacological model for cardiovascular research [35]. The body experiences oxidative stress when its antioxidant defense systems, which include glutathione peroxidase, catalase, and superoxide dismutase, are overwhelmed by the formation of reactive oxygen species (ROS). Cardiovascular diseases are related by this imbalance and its negative effects. The comparatively low antioxidant capacity within cardiac muscle cells may contribute to their heightened vulnerability to oxidative injury. One effective way to protect the heart from damage is by using pharmacological methods to reduce oxidative stress. This work utilized H9c2 cells as a pharmacological model [32]. The activity of these Ca^2+^ handling proteins is regulated by many signaling pathways, such as CaMKII, β-adrenoceptor (β-AR), protein kinase A (PKA), and PKC [36]. The apoptotic rate of H9c2 cells was measured at various SPN concentrations using annexin V/PI staining (Fig. 5B). Studies have demonstrated that quercetin (Que) can shield H9C2 cardiomyocytes from harm caused by hypoxia/reoxygenation (H/R) by reducing the activity of JNK and p38 signaling pathways. This regulation inhibits cell death by influencing Bcl-2, Bax, and caspase-3 expression either directly or indirectly [36]. A notable rise in the level of reactive oxygen species (ROS) was observed in H9c2 cells after exposure to hypoxia. The DCFH-DA images of the cells displayed that the hypoxia exhibit substantially greater levels of fluorescence. Flow cytometry analysis revealed that the level of reactive oxygen species (ROS), as measured by DCF fluorescence, was 42.17 percent higher in the hypoxia group compared to the normoxia group (19.23 ± 2.46 percent of normoxia, p < 0.001) has been reported [37]. For example, future atrial modeling studies would greatly benefit from delving further into the effects of oxidative stress, the β-adrenergic and CaMKII networks interact with each other and other pathways [38]. At 5 mmol/L or 10 μmol/L, dithiothreitol (DTT) or coenzyme Q10 (CoQ10) considerably reduced an increase in diastolic Ca^2+^ concentration induced by ATX-II, restored the amplitude of Ca^2+^ transients, and eliminated cellular arrhythmias. These findings point to the possibility that CaMKII activation is induced by ROS production by ATX-II [39]. The regulatory domain C290 site is one of three possible target sites suggested by the S-nitrosylation sites on CaMKII. The results point to a new CaMKII activation. Dysregulated cellular homeostasis progresses, the disease process accelerates, and dysregulation increases as a result of the CaMKII-cytosolic Ca^2+/^CaM interaction [40].

### 3.5 *In vivo* experiments in zebrafish model

Myocardial fibrosis is considered to be connected to the level of hypertrophy and can increase the risk of arrhythmias. The proper functioning of the heart depends on the accurate synchronization of contraction timing and heart rate in all areas. Alterations in excitation-contraction (EC) coupling, which encompass the initiation of contractile proteins by Ca^2+^ and the subsequent elimination of calcium to induce relaxation, may play a pivotal role in the progression of cardiac failure. Our *In vitro* findings with H9C2 cells indicated that the irregular heart rhythm induced by Phenanthrene (Phe) was linked to changes in intracellular calcium levels ([Ca^2+]^i). This was observed in ventricular myocytes by a reduction in sarcoplasmic reticulum calcium reuptake and noticeable calcium buildup in the cytoplasm following exposure to Phe [41]. Recording the ECG of adult zebrafish heart *In vivo*. Commonly used medicines like astemizole and terfenadine, known to lengthen human QT intervals, were discovered to likewise lengthen zebrafish QT intervals [42].

### 3.6 Experiments measuring the effects on the cardiovascular system

**Fig. 6:**
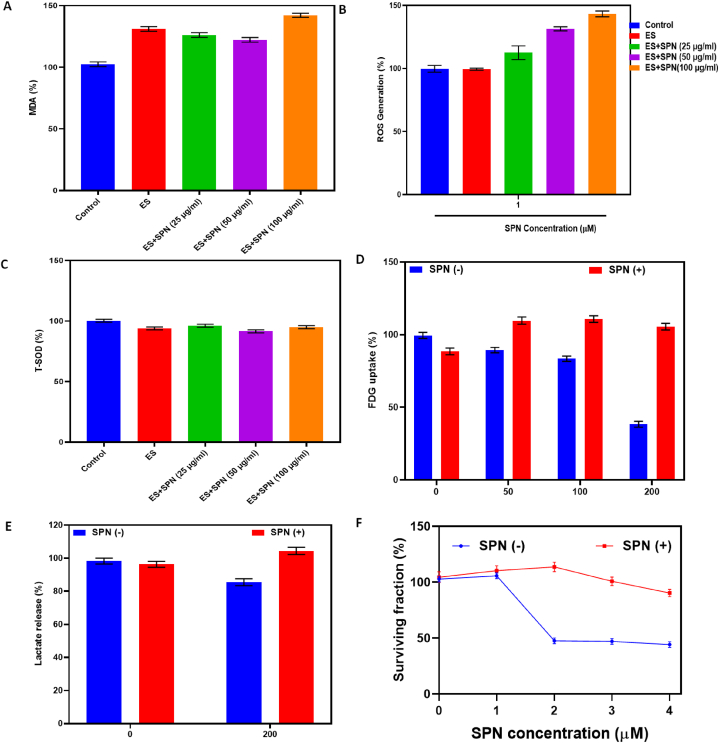
Measurement of malondialdehyde (MDA), reactive oxygen species (ROS), and total superoxide dismutase (T-SOD) in zebrafish following treatment with SPN and electrical stimulation. Values are expressed as mean ± SD. ∗p < 0.05 and ∗∗p < 0.01 vs. control (A,B and C). FDG uptake and lactate release in differentiated H9C2 cells after 24-h exposure to 200 μM SPN (D&E) Time course of reduced survival over 72-h exposure to graded doses of SPN. Data are the mean ± SE of values obtained from independent experiments. p < 0.005; ‡p < 0.001 compared with untreated controls (F).

In this study, quantification of malondialdehyde (MDA), reactive oxygen species (ROS), and total superoxide dismutase (T-SOD) in zebrafish after exposure to SPN and electrical stimulation. FDG absorption and lactate secretion in specialized H9C2 cells following a 24-h exposure to 200 μM SPN. The graph in Fig. 6(A–F) shows the decreasing survival rate over 72 h when exposed to varying levels of SPN. Similarly, the production of reactive oxygen species (ROS) in zebrafish treated with AC rose proportionally with the dosage. The ROS content was approximately 3 times greater in the 7.27 μM AC-treated group and approximately 6 times higher in the 8.23 μM AC-treated group compared to the control group. Following the administration of AC therapy, the impact of AC on SOD and MDA levels in larvae was assessed. The study revealed a substantial decrease in SOD activity in embryos treated with AC, while MDA levels were considerably elevated in groups treated with 7.27 and 8.23 μM of AC. a variety of concentrations that enabled survival and longitudinal investigations on behavior and heart rate. The administration of cadmium started at 24 h post-fertilization, following the establishment of the body axis and the beginning of primary neurogenesis and the production of monoaminergic cells [43]. The reported findings revealed that free fatty acids (FFA) led to a notable elevation in MDA levels P < 0.01 While MDA levels were reduced by all concentrations of Crocetin (CCT) compared to the 2.5 μM CCT group and compared to the 5 μM CCT and 10 μM CCT groups. There was a notable decrease in SOD and CAT P < 0.01 activity after administering FFA. These changes were reversed and the activity of both enzymes was significantly improved when treated with 5 μM and 10 μM CCT has been reported earlier [44]. Those zebrafish larvae subjected to 8 mg/L AZM showed strong green fluorescence (p < 0.01), in contrast to the control group that showed weak fluorescence. Markers of oxidative stress in organisms, such as MDA and GSH, are crucial [45]. Zheng et al., 2023 reported that, athycaltide-1 (ATH-1) and AIP inhibited the activation of CaMKII and ERK1/2 in hypertrophic cells. The results in vivo were in agreement with those in vitro suggesting that ATH-1 reduced ROS and inhibited the activation of CaMKII and ERK1/2, thereby attenuating Ang II-induced myocardial hypertrophy [46].

### 3.7 Quantification of cardiovascular gene expression

**Fig. 7:**
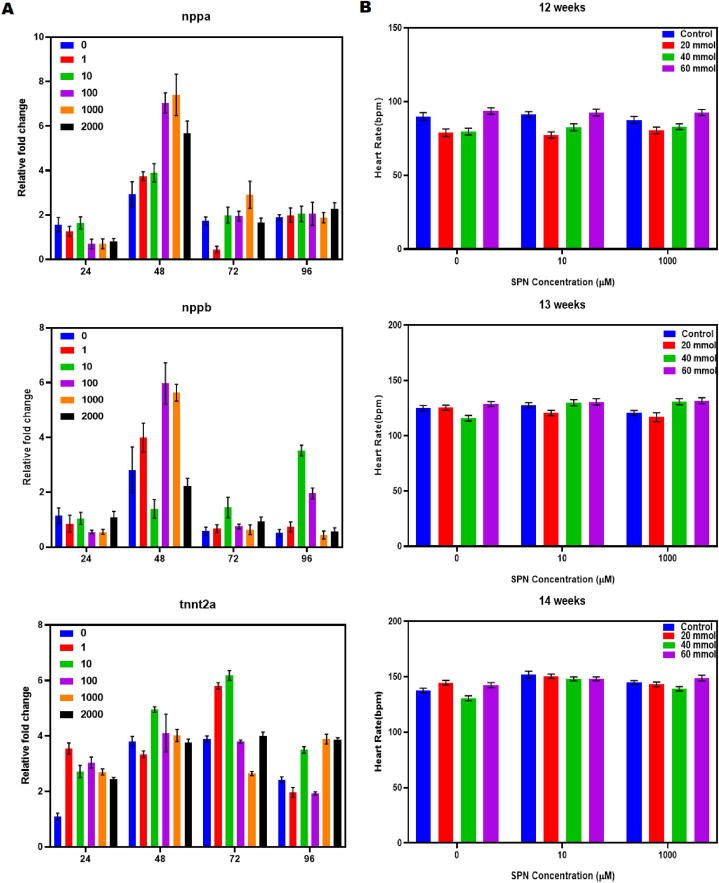
Quantification of cardiovascular gene expression levels (nppa, nppb, and tnnt2a) following treatment with SPN for 24, 48, 72, and 96 h, and subsequent normalization of the values. Gene expression is displayed as fold induction relative to controls at each time point. (A). Heart rate of zebrafish at 12, 13 and 14 weeks by SPN treatment. One-way ANOVA was used in analysis and data was expressed as mean ± SD from three independent biological replicates of at each. ∗p < 0.05, ∗∗p < 0.01, and ∗∗∗p < 0.001 compared to control groups (B).

Using the ABI Step One Plus RT-PCR system, qPCR tests were conducted. Evaluation of gene expression levels related to cardiovascular health (nppa, nppb, and tnnt2a) was conducted following SPN treatment for durations of 24, 48, 72, and 96 h, with subsequent normalization of the obtained data. Gene expression data are depicted as fold changes relative to control levels at each respective time point. Heart rate of zebrafish at 12, 13, and 14 weeks under SPN therapy (Fig. 7 A&B). The subsequent signaling pathways that control the expression of genes involved in cardiac development (nppa, nppb, tnnt2a) are impacted by this. To confirm or refute this hypothesis, additional experiments are required to investigate the SPN effects on ROS levels, CaMKII activation status, and the activity of transcription factors that regulate genes related to the heart. Likewise, total RNA was isolated from zebrafish larvae at 72 h post-fertilization that were treated with propranolol hydrochloride. Subsequently, a reverse transcription reaction was conducted. TBX5, a T-box transcription factor, plays a crucial role in controlling the development of the heart and the expression of genes. This led us to hypothesize that a TBX5-dependent pathway could be a potential explanation for the arrhythmia induced by Phe [41]. The calculated statistical significance (P) values for 100 μM and 1000 μM concentrations were 0.0012 and 0.0172, respectively, while for 2000 μM, the values were 0.0172. NPPB and NPPA are both used as biomarkers for myocardial stress. There was a statistically significant increase in Nppb expression in groups treated to dosages of P = 0.0077 at 48 hpf. following 96 h following fertilization, the groups treated with low doses of NEP exhibited higher levels of nppb expression than those treated with high doses (P = 0.0077 for 1 μM, P < 0.0001 for 10 μM). At 24 h post-flight, the expression of Tnnt2a, a gene associated with cardiac contractility, was significantly upregulated in all groups treated with NEP has been reported [47]. Hybridization with digoxin labels performed in situ *In vitro* transcription and polymerase chain reaction were used to create RNA probes. Zebrafish larvae were examined using in situ hybridization to identify changes in the expression of the β-catenin, parkin, and gfap genes [48]. Prominent expression was seen in both the atrial and ventricular chambers of the heart during development. Popdc2 was found in the heart of adults and showed a little expression in skeletal muscle [49]. Inducing nkx2.5 leads to the enlargement of the heart region and enhances the activation of genes related to the heart in zebrafish embryos. Previous research has shown that mutations in nkx2.5 cause significant abnormalities in heart development in mice and humans [50].

### 3.8 SPN performance in zebrafish heart rate

**Fig. 8:**
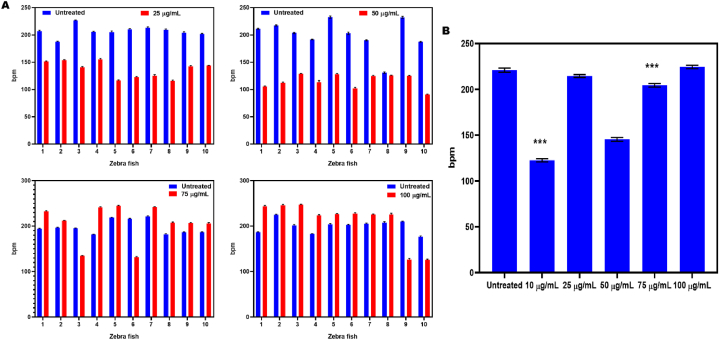
Identifying changes in heart rate in zebrafish following treatment with SPN that either decrease or enhance heart contraction rate. The heart rate of 10 zebrafish was monitored right before administering the medication (A). Note that the same response to drug administration was observed in all zebrafish Statistical analysis of zebrafish indicates that SPN regulates cardiac rate after expressing electrical stimulation (B). The experiments were expressed as mean ± SD. Three independent biological replicates were included. ∗p < 0.05, compared among groups.

Determining alterations in zebrafish heart rate after treatment with SPN that can either reduce or raise the pace of cardiac contractions. The heart rate of 10 zebrafish was observed prior to the drug being administered. It is important to note that all zebrafish showed a consistent response to drug treatment, showing that SPN controls heart rate following electrical stimulation (Fig. 8A and B). Based on the previous results of the cardiovascular toxicity test, the optimal dose of NEP to increase heart rate was 10 μM, whereas 1000 μM NEP caused only modest inhibition. Zebrafish at 20 h post fertilization were given these doses and monitored for an hour to see how they affected heart rate. At 45 hpf, larvae in 10 μM group showed a significantly increased heart rate (P = 0.0094), while larvae at 1000 μM NEP set required a somewhat stimulating effect on their heart rate. The cardiovascular effects of cocaine were successfully mitigated by the use of propranolol and other adrenergic antagonists [43]. Between 24 and 96 h post-fertilization (hpf), heart rate, pericardial area, cardiac function, and looping were measured in both wildtype and transgenic zebrafish embryos carrying the cmlc2 gene. Cardiotoxicity in zebrafish embryos was further analyzed at 96 hpf using MS-222, with heart rates measured according to specific protocols [51]. Cardiac arrhythmia was measured quantitatively by analysing 20-s video snippets taken from individual embryos. The onset of cardiac contraction was observed, and the video frame number was documented for each beat [41]. Numerous investigations have explored the medication's influence on heart rate under stress conditions. In our research, we observed a significant alteration in heart rate reversal in zebrafish larvae following exposure to a concentration of 2.5 μM SCH23390 for one day. This observation implies a potential involvement of D1R in modulating the effects induced by NEP at concentrations of 10 μm and 1000 μm. Additionally, the findings suggesting at that the larval D2 receptor may operate via a distinct mechanism compared to the D1 receptor when exposed to NEP [47]. AC exposure was observed to reduce the heart rate of zebrafish, which contradicts our findings. There are two potential explanations for this. The initial factor is the age gap among fish. In our work, zebrafish were subjected to alternating current (AC) from 4 to 96 h post-fertilization (hpf) using zebrafish embryos at 48 hpf [52]. With regard to treatment Embryos treated with verapamil and those not treated were immediately rinsed three times, 18 h after exposure. The experiment involved using either 1 mM NAC with 50 or 100 μM of verapamil and vehicle (DMSO). 2 hrs of following exposure, heart rates were measured [53].

### 3.9 SPN exposure to zebrafish

**Fig. 9:**
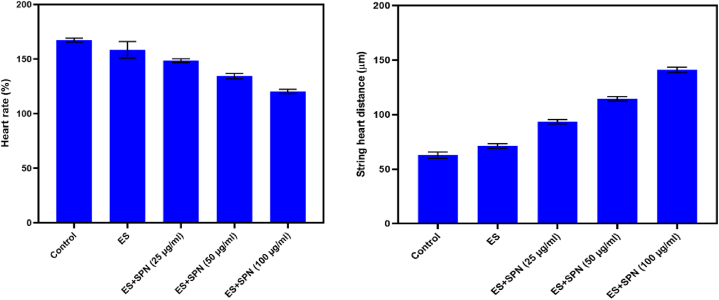
Impact of SPN exposure on zebrafish cardiac abnormalities, heart rate variability based on 100 μg/ml concentration, and string heart distance. ∗∗∗p < 0.0001 versus control.

The effects of SPN exposure on zebrafish cardiac defects, heart rate variability at a dose of 100 μg/ml, and string heart distance are shown in Fig. 9. Like the same results found in zebrafish embryos exposed to PM2.5 developed heart abnormalities such as bradycardia, irregular heartbeats, and pericardial edema. Embryos of zebrafish exposed to PM2.5 showed a steady increase in the length of the string and the area surrounding the heart (SV - BA) has reported [51]. It is crucial to establish if the higher rate of early death in SCN5A-D1275N zebrafish is caused by malignant bradyarrhythmias, tachyarrhythmias, or other reasons [54]. DNA repair during cardiac development in zebrafish, which includes controlling the expression of myocardial genes like nkx2.5 and producing an adequate number of myocardial precursors for normal cardiac morphogenesis [55]. M-mode offers a high temporal resolution to show the movement of the myocardium walls as they contract in systole and relax in diastole [56]. Fu et al., 2024 reported that, succinate worsened sepsis arrhythmias but decreased sepsis mortality and morbidity. In septic rats, succinate improves metabolic profiles and increases survival rates by increasing muscle oxygen consumption and decreasing reactive oxygen species (ROS) production [57]. At the same time, Larsen et al., 2023 reported that, Statistical analysis has not shown an elevated risk of our combined arrhythmia outcome, which encompasses cardiac arrest, any arrhythmia, or the need for a pacemaker to be implanted [58].

## 4 Conclusion

In summary, sophoridine (SPN), a natural compound derived from traditional Chinese herbs, demonstrates notable potential as an anti-arrhythmic agent with antioxidant properties. The study revealed SPN's ability to enhance cell viability in H9C2 rat cardiomyocytes, accompanied by dose-dependent increases in mitochondria-derived reactive oxygen species (ROS) as well as CaMKII signaling and calcium/calmodulin-dependent protein kinase II. Importantly, effects of SPN were mitigated by scavenging with ABTS and DPPH. Glucose fluctuations exacerbated ventricular arrhythmias induced by SPN via ROS/CaMKII pathway activation. Zebrafish experimental models supported SPN's regulatory effects on heart rate, alongside significant transcriptional deregulation of key heart development genes (nppa, nppb, tnnt2a). These findings highlight the multifaceted pharmacological properties of SPN and contributing capable paths for the development of novel anti-arrhythmic treatment approaches. However, a limitation of this study is the reliance on in vitro and zebrafish models, which may not fully replicate the complex physiological conditions in humans. Future technological advances, such as gene editing of CaMKII, offer promising outcomes. Further research is needed to assess the potential side effects, toxicity, and dosage of viral vectors. A deeper understanding of CaMKII distribution, isoforms, and mechanisms is essential for advancing drug development. Further research, including in vivo studies and clinical trials, is necessary to validate these findings and determine the therapeutic relevance of SPN in human cardiovascular health.

## Funding

This research was supported by Shanxi Provincial Health Commission Fund (2018030).

## Consent to participate

All of the authors agree to work on this project.

## Consent to publish

The approval of each author to publish the manuscript has been obtained. The manuscript is not currently under review by any other journal and has not been published before.

## Data availability statement

Data will be made available on request. All the data generated or analyzed during the study are included in this published article.

## CRediT authorship contribution statement

**Shuai Sun:** Writing – review & editing, Supervision, Project administration. **Fangdi Shi:** Writing – original draft, Resources, Methodology, Investigation. **Gang Zhao:** Validation, Formal analysis, Data curation. **Hong Zhang:** Validation, Formal analysis, Data curation.

## Declaration of competing interest

The authors declare the following financial interests/personal relationships which may be considered as potential competing interests: There are no conflicts of interest for the present research work. If there are other authors, they declare that they have no known competing financial interests or personal relationships that could have appeared to influence the work reported in this paper.
